# Supplementary material for: Combination Treatment of a Phytochemical and a Histone Demethylase Inhibitor—A Novel Approach towards Targeting TGFβ-Induced EMT, Invasion, and Migration in Prostate Cancer
Source: Int J Mol Sci. 2023 Jan 17;24(3):1860. doi: 10.3390/ijms24031860 (PMC9915876; doi:10.3390/ijms24031860)
Supplement: Supplementary file 1 [file ijms-24-01860-s001.zip › ijms-2112611-supplementary.pdf]

Supplementary file 2

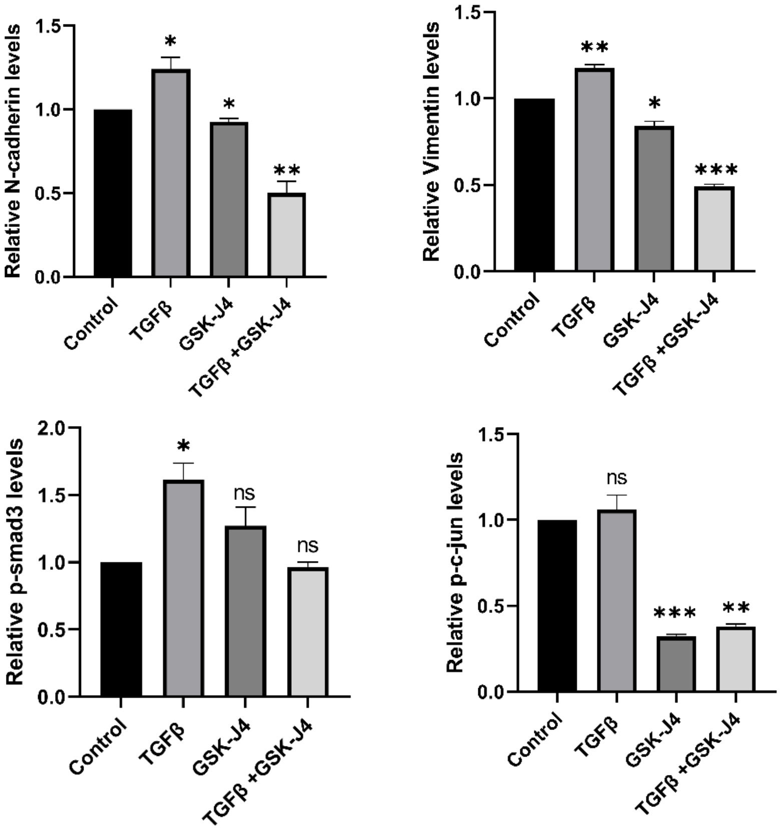

Figure S1. Western blot quantification using ImageJ software for blot images on fig 1(d), *p<0.05, **p<0.01, ***p<0.001

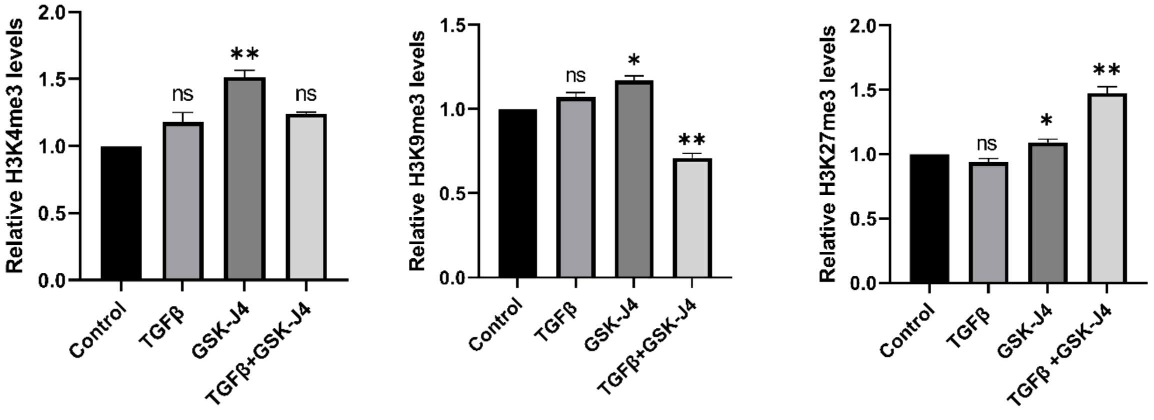

Figure S2. Western blot quantification using ImageJ software for blot images on fig 1(g), *p<0.05, **p<0.01, ***p<0.001

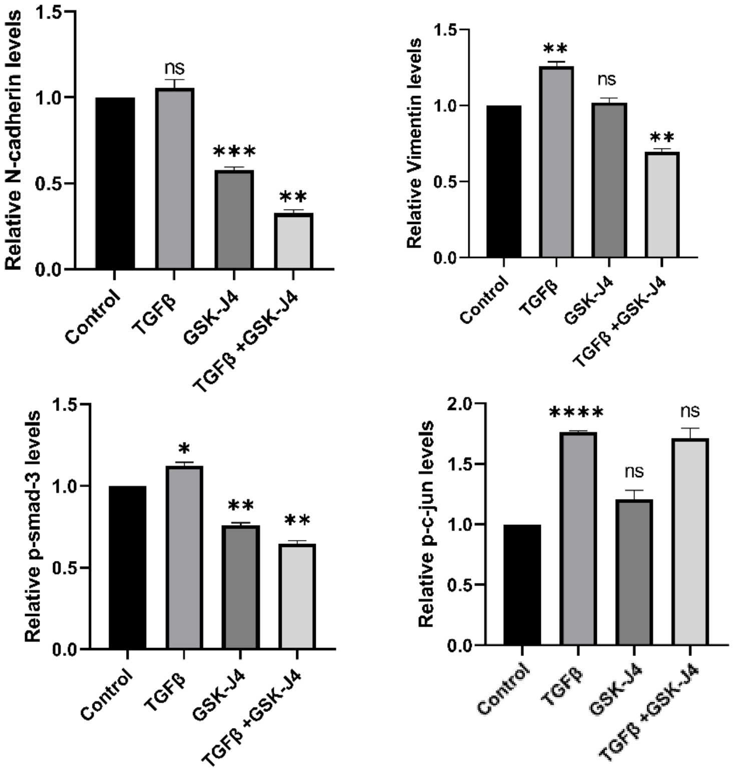

Figure S3. Western blot quantification using ImageJ software for blot images on fig 2(b), *p<0.05, **p<0.01, ***p<0.001, ***p<0.0001

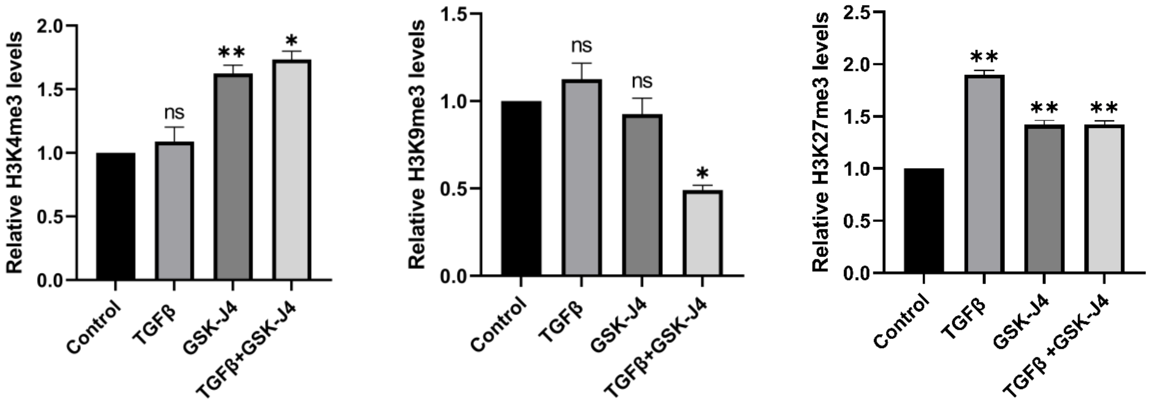

Figure S4. Western blot quantification using ImageJ software for blot images on fig 2(e), *p<0.05, **p<0.01, ***p<0.001

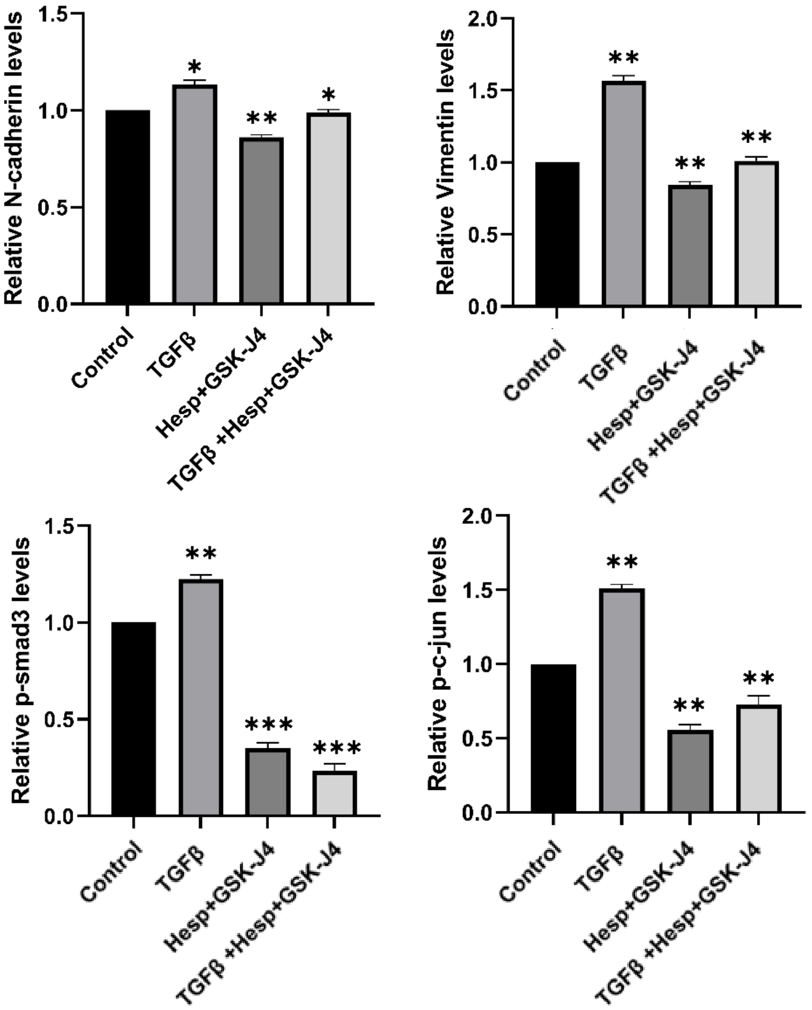

Figure S5. Western blot quantification using ImageJ software for blot images on fig 3(c), *p<0.05, **p<0.01, ***p<0.001

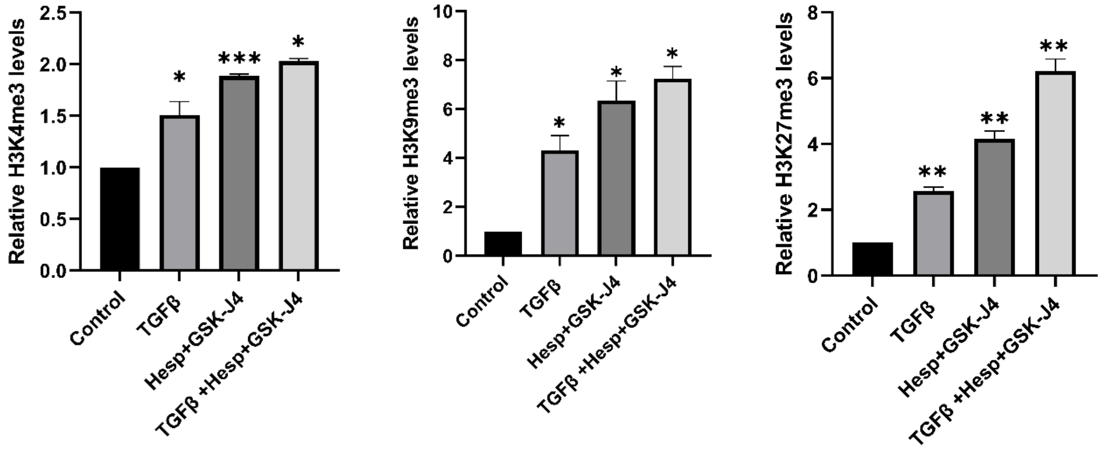

Figure S6. Western blot quantification using ImageJ software for blot images on fig 3(f), *p<0.05, **p<0.01, ***p<0.001

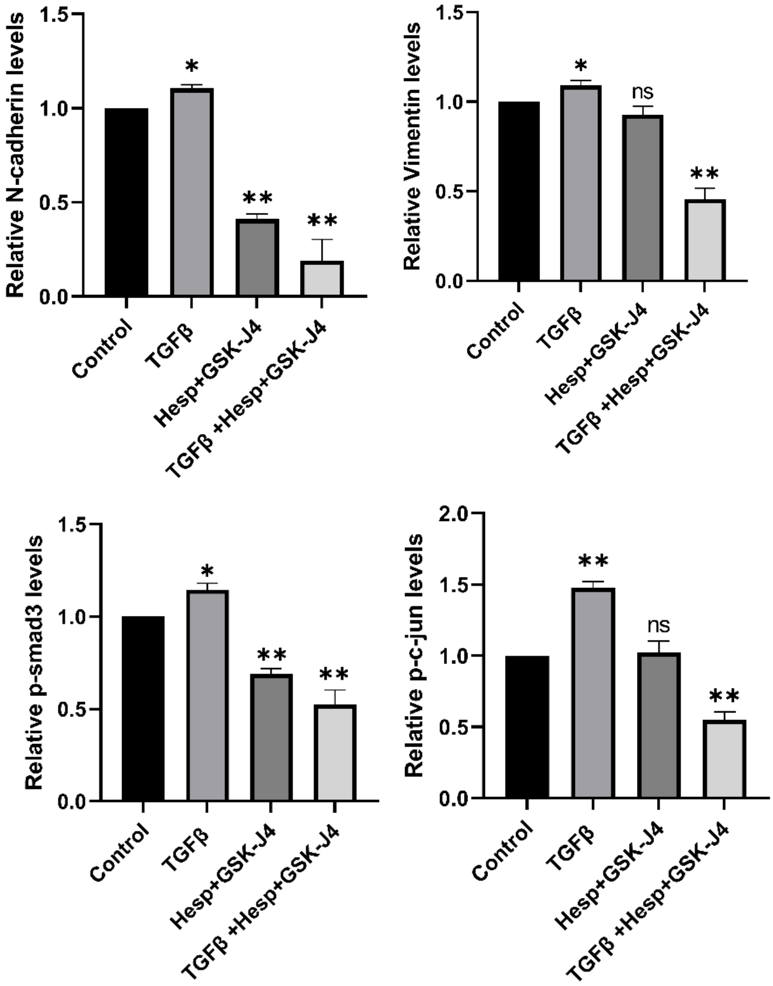

Figure S7. Western blot quantification using ImageJ software for blot images on fig 4(c), *p<0.05, **p<0.01, ***p<0.001

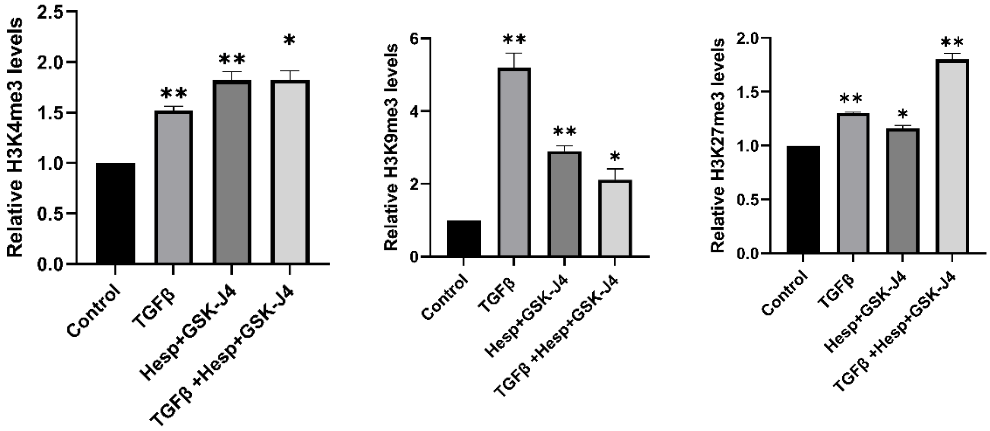

Figure S8. Western blot quantification using ImageJ software for blot images on fig 4(f), *p<0.05, **p<0.01, ***p<0.001
